# PD136 Re-evaluation Of Chest X-Ray Screening For Lung Cancer With Consideration Of Study Context

**DOI:** 10.1017/S0266462324003738

**Published:** 2025-01-07

**Authors:** Chisato Hamashima, Teruhiko Terasawa, Yuki Kataoka, Keisuke Anan, Satoyo Hosono

## Abstract

**Introduction:**

Lung cancer causes a heavy burden worldwide and an efficient screening program is needed. Although low dose computed tomography screening has become mainstream for lung cancer screening, chest X-ray (CXR) screening has continued in Japan. We re-evaluated the efficacy and effectiveness of CXR screening and reconsidered the context of the studies.

**Methods:**

We performed a systematic review and meta-analysis of CXR screening for lung cancer. The study design included randomized controlled trials (RCTs), cohort studies, and case-control studies (CCSs) that evaluated the efficacy or effectiveness of CXR screening. Searches were conducted in the PubMed, Cochrane Library, Web of Science, and Ichushi-Web databases for literature published up to April 2022. We examined the settings of the selected studies.

**Results:**

From about 4,000 candidate articles, six RCTs, one cohort study, and six CCSs were selected. Five RCTs were conducted in the 1960s and 1970s, except for the Prostate, Lung, Colorectal, and Ovarian Cancer (PLCO) Screening Trial. Six CCSs conducted in Japan reported reductions in mortality from lung cancer. A meta-analysis of the six CCS showed a 47 percent reduction in mortality from lung cancer (adjusted odds ratio 0.53, 95% confidence interval: 0.50, 0.63). In the PLCO trial, mortality was reduced by nine percent at the six-year follow up, but this result was not statistically significant. The histological distribution of lung cancer was similar between the PLCO trial and the Japanese CCSs.

**Conclusions:**

The dilution effect might affect the PLCO trial results because of extended follow up beyond the lead time of CXR screening. Although evidence on CXR screening for lung cancer is limited, CXR screening might be adopted in Japan considering the histological changes in lung cancer that have occurred due to the decline in smoking rate.

